# Transient Myocarditis Associated with Fulminant Colitis

**DOI:** 10.5402/2011/652798

**Published:** 2011-06-01

**Authors:** J. M. L. Williamson, R. S. J. Dalton

**Affiliations:** Department of Surgery, Musgrove Park Hospital, Tauntonl, Somerset, TA1 5DA, UK

## Abstract

*Case Summary.* An 18-year old man presented with a three-week history of abdominal pain, weight loss and bloody diarrhoea. He was profoundly septic, with generalised abdominal tenderness. CT and flexible sigmoidosopy confirmed colitis of the colon with rectal sparing. Laparotomy was performed when conservative management failed to improve his condition. Subtotal colectomy, with end ileostomy and mucus fistula formation, was performed in light of active colitis. Despite successful operative intervention the patient acute left ventricular failure, raising the possibility of giant cell myocarditis, which fully resolved before a definitive diagnosis could be reached. *Discussion.* It is possible that the transient cardiac failure in this case may represent an overwhelming inflammatory response or myocarditis. Inflammatory bowel disease is rarely associated with giant cell myocarditis (GCM). GCM usually affects a young population and its prognosis is variable, ranging from complete recovery, remission with recurrence and fatality. The management of this group of patients is still relatively experimental. *Conclusion.* Fulminant colitis can be associated with a rapid deterioration in cardiac function. Causes include sepsis, systemic inflammatory response syndrome or myocarditis. GCM should be considered in patients with new onset of left ventricular failure that decline rapidly.

## 1. Case Report

A previously well 18-year-old carpet fitter presented with a three-week history of progressive abdominal pain, anorexia, and 10 Kg weight loss. There was associated diarrhoea, with blood and mucus mixed with stool, nausea, and vomiting. He denied any pyrexia or rigors, foreign travel, or ingesting any unusual food. There was no significant medical history or family history. On examination the patient was severely dehydrated, cahexic, hypotensive, and tachycardic. Cardiovascular examination was otherwise unremarkable and a soft, nonperitonitic abdomen with generalised tenderness was detected. The abdomen was not distended and no organomegaly was identified. Rectal examination detected no abnormality, except for a small perianal fistula. A diagnosis of severe dehydration secondary to either colitis or gastroenteritis was made, and aggressive fluid resuscitation commenced.

The patient's condition deteriorated after aggressive fluid resuscitation (five litres of crystalloid fluid over 2 hours). He remained in shock (pulse 160 bpm, blood pressure 86/50 mm Hg) and became tachypnoeic (respiratory rate 50) and hypoxic (saturations 83% on air). Repeat examination revealed an elevated jugular venous pulse (6 cm) and widespread wheeze throughout both lung fields. Some diffuse lower abdominal tenderness was again seen, but no evidence of distension or peritonism. Haematological investigations revealed an elevated CRP, but normal white cell count and lactate. Arterial blood gas confirmed hypoxia in addition to compensated metabolic acidosis. Pulmonary oedema was seen on a chest radiograph, and an echocardiogram showed right bundle branch block.

**Figure 1:**
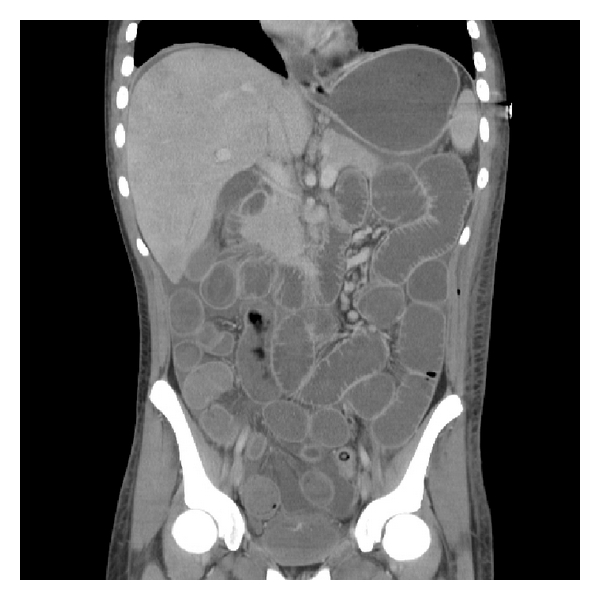
Abdominal CT showing multiple oedematous and dilated loops of both small and large bowel.

It was thought that the patient might have colitis with systemic inflammatory response syndrome causing acute pulmonary oedema. The patient's respiratory signs improved with frusemide and associated diuresis, but his tachycardia and hypotension persisted. Ionotropic support and noninvasive oxygen therapy commenced, although his respiratory effort declined and he was subsequently ventilated. After a period of stabilisation an ultrasound was performed, which noted some thickening of the caecal wall, which was confirmed with abdominal computed tomography (CT), which also identified distended loops of both large and small bowel (Figure 1). A flexible sigmoidoscopy confirmed an oedematous mucosa (consistent with inflammatory bowel disease), with rectal sparing. Multiple biopsies were taken.

Conservative and interventional management options were considered. It was thought that the medical management of the colitis would not prove successful given the rapid decline in the patient's condition. Operative intervention to remove the affected bowel was thought to be the most beneficial option. At laparotomy active colonic colitis was confirmed and copious clear peritoneal fluid seen, although there was no perforation. A subtotal colectomy with end ileostomy and mucus fistula formation was performed. It was thought that this was an acute inflammatory process and probably due to ulcerative colitis, with a possibility of superimposed infection. Histological examination of the resected specimen identified fulminant colitis with a cobblestone mucosa and inflammation involving the mucosa and submucosa. In addition, pseudopolyps, cryptitis, and crypt abscesses were noted, but no granulomata or features of pseudomembranous colitis seen. Subsequent viral serologies (including influenza, adenovirus, lyme disease, and yersinia) were all negative.

Postoperatively the patient continued to require ionotropic support. A transthoracic echocardiography showed left ventricular failure, with an ejection fraction of 20%. Cardiology review hypothesised that the poor cardiac function could be due to inflammatory disease causing giant cell myocarditis, and it was thought that this had a very high mortality. Management options were again reassessed, with cardiac biopsy and a heart-lung transplant considered. However, the patient's condition improved with supportive management. His cardiac failure resolved, and repeat echocardiography at 11 days showed that left ventricular function had returned to normal. After full recovery he was discharged, and he awaits further cardiology review.

## 2. Discussion

Myocarditis (cardiac muscle inflammation) is associated with symptoms of congestive cardiac failure, chest pain, arrhythmias, syncope, and sudden death [1–3]. Myocarditis can be broadly classified into infective and noninfective causes. Infective causes include viral (both DNA and RNA), bacterial, mycobacterial, fungal, protozoal, rickettsi, chlamydiae, and parasitic [2]. Noninfective mechanisms include environmental factors (toxins, alcohol, and chemotherapy), metabolic abnormalities or, more frequently, immunological factors [2]. 

Inflammatory bowel disease is uncommonly associated with cardiac disease [1–3]. These extraintestinal manifestations include pericarditis, pericardial effusion, myocarditis, myocardial infarction, endocarditis, and arrhythmias [1, 3]. Although rare, myocardial inflammation related to inflammatory bowel disease is normally associated with the presence of multinucleate giant cells [1–3].

The presence of inflammatory bowel disease and cardiac dysfunction in this patient is therefore highly suggestive of GCM, although a cardiac biopsy was not performed to confirm diagnosis. GCM is a poorly understood disease, and it can be idiopathic in origin or associated with autoimmune disorders and inflammatory bowel disease [1, 3, 4]. GCM usually affects a young population and its prognosis is variable, ranging from complete recovery, remission with recurrence, and fatality [1, 3, 4]. The majority of patients have a rapid fatal decline after presentation [1, 3–5]. The management of this group of patients is still relatively experimental. Triple immunosuppression therapy and cardiac transplantation has been recommended [4, 5]. However, there have been cases of recurrence of GCM in cardiac transplants [4].

## 3. Conclusion

This case highlights how fulminant colitis can be associated with a rapid deterioration in cardiac function. The cause of this patient's transient phenomena is unclear and could either be due to an overwhelming systemic inflammatory response syndrome or to a myocarditis. Resection of the affected segment of bowel proved an effective treatment, resulting in the restoration of cardiac function. Giant-cell myocarditis (GCM) should be considered in patients with new onset of left ventricular failure that decline rapidly [1].
